# Large scale patterns in mussel beds: stripes or spots?

**DOI:** 10.1007/s00285-018-1293-z

**Published:** 2018-09-05

**Authors:** Jamie J. R. Bennett, Jonathan A. Sherratt

**Affiliations:** Department of Mathematics and Maxwell Institute for Mathematical Sciences, Heriot-Watt University, Edinburgh, EH14 4AS UK

**Keywords:** Mussels, Mussel–algae interaction, Activator-inhibitor, Self-organisation, Pattern formation, Turing–Hopf bifurcation, Two-dimensional stability, Tranverse perturbations, 35Q92, 35K57, 35B36, 35C07

## Abstract

An aerial view of an intertidal mussel bed often reveals large scale striped patterns aligned perpendicular to the direction of the tide; dense bands of mussels alternate periodically with near bare sediment. Experimental work led to the formulation of a set of coupled partial differential equations modelling a mussel–algae interaction, which proved pivotal in explaining the phenomenon. The key class of model solutions to consider are one-dimensional periodic travelling waves (wavetrains) that encapsulate the abundance of peak and trough mussel densities observed in practice. These solutions may, or may not, be stable to small perturbations, and previous work has focused on determining the ecologically relevant (stable) wavetrain solutions in terms of model parameters. The aim of this paper is to extend this analysis to two space dimensions by considering the full stripe pattern solution in order to study the effect of transverse two-dimensional perturbations—a more true to life problem. Using numerical continuation techniques, we find that some striped patterns that were previously deemed stable via the consideration of the associated wavetrain solution, are in fact unstable to transverse two-dimensional perturbations; and numerical simulation of the model shows that they break up to form regular spotted patterns. In particular, we show that break up of stripes into spots is a consequence of low tidal flow rates. Our consideration of random algal movement via a dispersal term allows us to show that a higher algal dispersal rate facilitates the formation of stripes at lower flow rates, but also encourages their break up into spots. We identify a novel hysteresis effect in mussel beds that is a consequence of transverse perturbations.

## Introduction

**Fig. 1:**
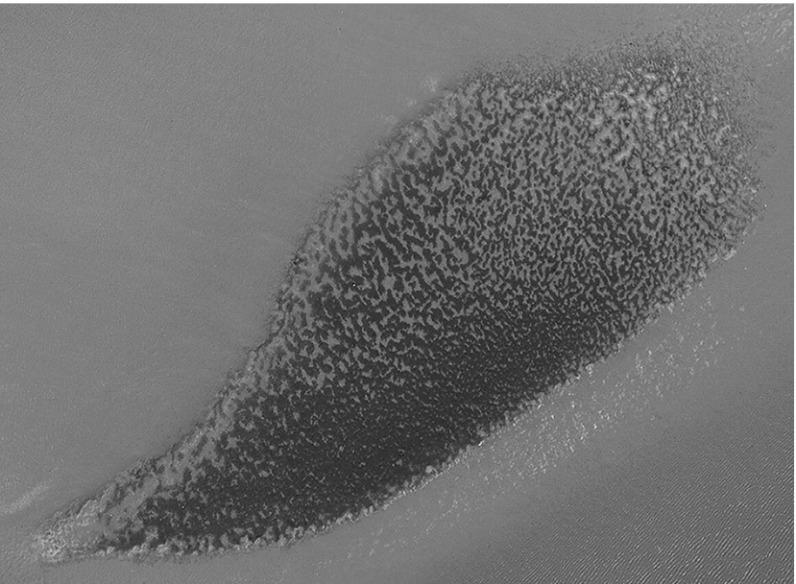
An image of large scale patterning in a mussel bed in the Dutch Wadden Sea. The image was taken from a topological map featured on BAG viewer, an interactive map powered by Kadaster, and is reproduced under the licence CC-BY Kadaster (https://creativecommons.org/licenses/by/4.0/legalcode)

Blue mussels (*Mytilus edulis*) are often known as the common mussel because of their persistence in abundance across various intertidal regions. They usually play important roles in biodiverse ecosystems as major food sources for aquatic and terrestrial animals, and also form the foundations of many shallow water, benthic habitats. One important role is the circulation of nutrients via filter feeding—water is siphoned over the gills where suspended biomass, such as algae, enters the digestive system. The excrements provide nutrients for other marine animals, and bi-products (pseudofaeces) become a form of enriched sediment, thought to increase species diversity (Dame et al. 1991). A comprehensive overview of the blue mussel has been collated in an online archive (Tyler-Walters 2008) by The Marine Biological Association. The ecological and agricultural significance of the blue mussel has prompted numerous empirical (Christensen et al. 2015; Dobretsov 1999; Capelle et al. 2014; Okamura 1986; Hughes and Griffiths 1988; Guiñez and Castilla 1999), as well as mathematical (van de Koppel et al. 2005; Liu et al. 2012; Sherratt 2016; Cangelosi et al. 2015; Ghazaryan and Manukian 2015; Holzer and Popović 2017), studies on mussel aggregation. Though sessile for the majority of their lives, individuals are able to reposition themselves—they anchor onto substrate by extending new byssus threads, which are shortened so that the main body of the mussel is dragged into position. Young mussels are particularly mobile, often settling away from older mussels to limit competition for food (Newell 1989), and, collectively, forming large beds on soft sediment by adhering to one another and ocean debris. Their local movement creates the opportunity for self organisation into large scale patterns. In this paper, we focus on periodic striped patterns in soft sediment mussel beds, observed in both the Dutch Wadden Sea (van de Koppel et al. 2005) and the Menai Strait (UK) (Gascoigne et al. 2005). We demonstrate how mussels can reorganise themselves from striped formations into spotted, patchy formations when the bed is subject to ecological change, providing insight into the origin of patterns such as that shown in Fig. 1, which is an aerial photograph taken over the Wadden Sea.

In this paper, we study a mathematical model based on the “reduced losses” hypothesis for striped mussel beds, which was proposed by van de Koppel et al. (2005). Mussels adopt a “strength in numbers” approach by forming dense aggregations to reduce their dislodgement by waves, and defend against predation. Soft-sediment beds are heavily influenced by the algal concentration in the benthic boundary layer, and this is the limiting factor for mussel growth (Dolmer 2000; Øie et al. 2002). Therefore, tidal currents play a significant role in pattern formation: the algal supply is simultaneously depleted and transported with the tide, inducing a long range inhibition between mussels. This, coupled with a short range activation to reduce losses, is the origin of periodic striped patterns, whereby a balance is struck between cooperation and competition between individual mussels. Before proceeding, we comment that there is an alternative “sediment accumulation” hypothesis for stripe formation in mussel beds, proposed by Liu et al. (2012).

We analyse an extended version of the original reduced losses model van de Koppel et al. (2005)—the non-dimensionalised equations for algal and mussel densities, *a*(*x*, *y*, *t*) and *m*(*x*, *y*, *t*), respectively, are given by 1a1b The parameters in these equations depend on a number of dimensional parameters (see van de Koppel’s paper 2005 for details) but convenient interpretations are as follows: $\alpha >0$ is the renewal rate of algae; $\beta$ is the tidal flow rate; $\delta >0$ is the scaled potential growth rate of mussels; $\mu >0$ is the maximal mussel mortality rate. In this paper we analyse (1) in both one space dimension (*x*, *t*) and two space dimensions (*x*, *y*, *t*). The *x*-coordinate is parallel to the direction of advection, with the *y*-coordinate acting perpendicular—in the direction of the shore. We take $\beta$ to be constant on the basis that the influx of algae with the incoming tide is the dominant effect; it would be more realistic to allow for oscillations in $\beta$, but doing so is significantly more complicated mathematically (Sherratt and Mackenzie 2016). However, we do take $\beta$ as a control parameter in our prospective numerical analysis, which is therefore relevant for slowly varying $\beta$. We allow $\beta$ to have either sign reflecting the bi-directional nature of tidal flow, so that sign changes in $\beta$ correspond to the tide changing direction at either high or low tide. Note however that (1) is unchanged by changes in the signs of $\beta$ and *x*. The parameter $\nu \gg 1$ is a ratio of algal and mussel dispersal rates. In the original reduced losses model (van de Koppel et al. 2005) algal movement is solely described by an advection term that mimics tidal flow and the transport of algae along with it. Cangelosi et al. (2015) extended the model by assuming a random movement of algae represented by a diffusive term, but their subsequent work focused on the special case where $\beta =0$. Equation (1) is the extended version of the reduced losses model, including both transport and dispersal terms.

We can study pattern formation by considering pattern solutions of (1). Mathematically, we do this by changing coordinate system to a frame of reference that moves in the direction of the pattern. This allows (1) to be reduced to a set of ordinary differential equations that are more easily analysed. In the real world, mussel beds are subject to disturbances, which we can model by adding small perturbations to our solutions. We are interested in determining which striped patterns are stable to these small disturbances, since they will persist in the disorderly and changeable setting of real mussel beds. For simplicity, we can categorise perturbations as either 1D—acting in the direction of water flow, or, 2D—acting, additionally, in a direction parallel to the shoreline. Previous work (Wang et al. 2009; Sherratt 2013a) has focused on the effects of 1D disturbances and the determination of ecologically relevant patterns by means of analysing (1), though we are unaware of any advances that consider stability to both 1D and 2D perturbations—a more accurate representation of the real world problem. Specifically, we study how the flow rate and algal dispersal rate affect stability. We pose the question; of those striped patterns that are stable to 1D disturbances, which are stable to 2D disturbances, and what is the fate of the 2D unstable patterns? We use numerical continuation techniques to determine those 1D striped patterns that will persist in their 2D setting, and verify that regular spotted patterns arise from those that are 2D unstable; we do this through numerical simulation of (1). In all numerical simulations we solve (1) on a unit square with periodic boundaries; utilising a spectral method (we used the fft2 and ifft2 routines from the Python library, NumPy) to remove the stiffness associated with diffusion terms, and an exponential time-differencing Runge-Kutta scheme which is described in Cox and Matthews (2002). Our work builds on a study of the stability of banded vegetation patterns observed in semi-arid desert regions by Siero et al. (2015).

In Sect. 2 we discuss the necessary conditions for the formation of striped patterns from a homogeneous steady state of (1). The remainder of the paper focuses on the stability of existing striped patterns and in Sect. 3 we detail a numerical methodology for testing 2D stability of striped mussel beds. In particular we produce a graphical representation of the rationale behind the process which we implement in Sect. 4 to obtain results about how tidal flow affects stability. In particular we identify a new type of hysteresis in the model that is a consequence of transverse 2D perturbations and we confirm this in numerical simulations of (1). The meaning of the term ‘hysteresis’ varies among authors; we use it to mean that the model solution has a dependency on its history, i.e. a change in state of the system due to a parameter decrease (increase) is non-reversible with a subsequent parameter increase (decrease) back to its initial value. In Sect. 5 we discuss the ecological implications of our findings.

## Onset of striped patterns in the extended reduced losses model

Equations (1) have two homogeneous steady states; an algae only steady state, $(a,m)=(1,0)$; and a co-existence steady state,

2 $$\begin{aligned} (a_s, m_s)=\left( \frac{\mu - \delta \alpha }{\delta (1-\alpha )}, \frac{\alpha (\delta - \mu )}{\mu - \delta \alpha }\right) , \end{aligned}$$

which we require to be positive. For the ecologically relevant parameters used in our study (1,0) is a saddle point, and therefore not of interest when considering the pattern forming tendencies of the model—we focus on (2). Mathematically, pattern solutions of (1) arise through a Turing–Hopf bifurcation (Turing bifurcation when $\beta =0)$. For this, we require (2) to be stable against homogeneous perturbations, and unstable to heterogeneous perturbations. A simple sufficient condition for (2) to be positive and stable to homogeneous perturbations is

3 $$\begin{aligned} 4>\delta>\mu >\delta \alpha \end{aligned}$$

(see Sherratt and Mackenzie (2016) for a detailed explanation).

**Fig. 2:**
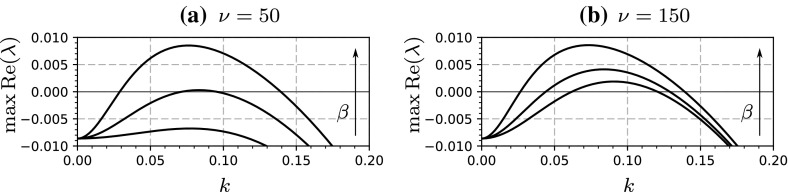
Pattern formation in (1) with (5) for two values of algal dispersal rate, $\nu$. We calculate the dispersion relation given by the determinant of (4) and plot the maximum real part of the eigenvalues, $\lambda$, as a function of wavenumber, *k*. We show plots for different tidal flow rates, $\beta =0,10,20$, in both panels, with the arrow denoting increasing $\beta$. In **(a)**, a critical $\beta$ exists that marks the onset of pattern formation, before which, no patterns will be observed; in **(b)**, pattern formation is independent of $\beta$ and occurs for all values. Note that due to symmetry, onset of patterns for $\beta <0$ is equivalent

At this point, we mention that we are able to neglect 2D perturbations when determining the onset of pattern formation. Siero et al. (2015) proved, for a general class of systems that includes (1), that primary destabilisation of (2) occurs for perturbations that are constant in the *y*-direction. Consequently, these perturbations grow quickest and, with our assumption of a supercritical Turing–Hopf bifurcation, form a striped pattern perpendicular to the direction of advection. Hence, we take $\varDelta = \partial ^2/\partial x^2$, since the onset of patterns in 1D is identical to the onset of patterns in 2D. We perform a Turing analysis by linearising (1) about (2), and substituting $(a-a_s,m-m_s)={\text {exp}}(ikx)(\tilde{a},\tilde{m})$, which yields an equation of the form, $(\partial /\partial t)(\tilde{a},\tilde{m})^T=M(\tilde{a},\tilde{m})^T$, where

4 $$\begin{aligned} M = \left( \begin{array}{c@{\quad }c} - \alpha - m_s + i\beta k - \nu k^2 &{} -a_s \quad \\ \delta m_s &{} \delta a_s - \mu /(1+m_s) - \mu m_s/(1+m_s)^2- k^2 \end{array} \right) , \end{aligned}$$

and *k* is the wavenumber of perturbations in the *x*-direction. Non-trivial solutions require ${\text {det}}(M-\lambda I)=0$, and this gives the decay rate of perturbations, $\lambda$, as a function of the various model parameters and *k*. In previous studies, it was shown that the onset of pattern formation occurs at a critical flow rate. In the extended model this is the case for low values of algal dispersal as seen in Fig. 2a, however for larger values pattern formation is independent of flow rate, and patterns exists for all $\beta$, as seen in Fig. 2b.

Thus, we can determine the origin of striped patterns in terms of parameters. For the remainder of this paper, we aim to study how they are affected by tidal flow and algal dispersal, and so we fix the parameters $\alpha$, $\delta$ and $\mu$; specifically

5 $$\begin{aligned} \alpha =0.6667,~~\delta =0.15,~~\mu =0.1333. \end{aligned}$$

These parameters were calculated by Wang et al. (2009), based on estimates of their constituent ecological quantities, and satisfy condition (3), giving a co-existence state that is stable to homogeneous perturbations. Striped patterns may then be generated but they may not be stable; in the subsequent sections we aim to show how $\beta$ and $\nu$ affect their stability.

## Methodology

Through a Turing–Hopf instability of the homogeneous steady state, we explained the origin of striped patterns in Sect. 2. Such solutions may or may not be stable in their own right, and consequently we now focus on the stability of the heterogeneous striped pattern solution. The flow rate, $\beta$, is likely to be the most variable parameter of (1), as it reflects the periodic advancing and receding of the ocean. Accordingly, we make $\beta$ our primary concern by selecting it as a control parameter, with the aim of establishing how stability changes when $\beta$ is varied. We can assess how the dispersal rate of algae affects stability by repeating the methodology described in this section for different values of $\nu$.

In this section, we first review 1D stability by taking $a=a(x,t)$, $m=m(x,t)$ and $\varDelta = \partial ^2/\partial x^2$ in (1). We shall see how the results can be used as a starting point for our main calculation of 2D stability in Sect. 3.2. For simplicity, we transform to a moving frame of reference $\xi =x-ct$, where *c* is the speed of the migrating pattern. Travelling waves, $a(x,t)=A(\xi )$ and $m(x,t)=M(\xi )$, of (1) are then solutions of

6a $$\begin{aligned} 0&= \nu \frac{\text {d}^2 A}{\text {d} \xi ^2} + (c+\beta )\frac{\text {d} A}{\text {d} \xi } + f(A,M) \end{aligned}$$

6b $$\begin{aligned} 0&= \frac{\text {d}^2 M}{\text {d} \xi ^2} + c\frac{\text {d} M}{\text {d} \xi } + g(A,M). \end{aligned}$$

Note this rescaling yields an advection-diffusion equation, with advection terms now featuring in both component equations of (6). By assuming a travelling wave solution form, we can automatically impose the boundary condition $A(L)=A(0)$, $M(L)=M(0)$, where *L* is the wavelength. Therefore, without loss of generality, we take our domain length to include one period of the solution by letting $0<\xi <L$. Travelling wave solutions now depend not only on $\beta$, but also on *c*. For each fixed value of $\beta$, a family of limit-cycle solutions (i.e. periodic patterns) exist beyond a critical value of *c*.

### 1D stability: the spectrum

In one space dimension, striped pattern solutions of (1) are periodic travelling waves and the associated stability problem is equivalent to that of the travelling wave solutions of (6). In general, to assess the (linear) stability of a solution, one must first apply small, spatio-temporal perturbations. In our moving frame of reference solutions of (6) are time-independent solutions of

7a $$\begin{aligned} \frac{\partial \hat{a}}{\partial t}&= \nu \frac{\partial ^2 \hat{a}}{\partial \xi ^2} + (c+\beta )\frac{\partial \hat{a}}{\partial \xi } + f(\hat{a},\hat{m}) \end{aligned}$$

7b $$\begin{aligned} \frac{\partial \hat{m}}{\partial t}&= \frac{\partial ^2 \hat{m}}{\partial \xi ^2} + c\frac{\partial \hat{m}}{\partial \xi } + g(\hat{a},\hat{m}), \end{aligned}$$

where $\hat{a}=\hat{a}(\xi ,t)$, $\hat{m}=\hat{m}(\xi ,t)$. For small $\bar{a}(\xi )$ and $\bar{m}(\xi )$, substitution of the perturbed travelling wave solutions

8 $$\begin{aligned} \hat{a}(\xi ,t)=A(\xi ) + \bar{a}(\xi ){\text {e}}^{\lambda t},\quad \hat{m}(\xi ,t)=M(\xi ) + \bar{m}(\xi ){\text {e}}^{\lambda t}, \end{aligned}$$

into (7), applying (6) and subsequently linearising about $A(\xi )$, $M(\xi )$ gives the eigenfunction equations,

9a $$\begin{aligned} \lambda \bar{a}&= \nu \frac{\partial ^2 \bar{a}}{\partial \xi ^2} + (c+\beta )\frac{\partial \bar{a}}{\partial \xi } + \bar{a}\frac{\partial f}{\partial a}(A,M) + \bar{m}\frac{\partial f}{\partial m}(A,M), \end{aligned}$$

9b $$\begin{aligned} \lambda \bar{m}&= \frac{\partial ^2 \bar{m}}{\partial \xi ^2} + c\frac{\partial \bar{m}}{\partial \xi } + \bar{a}\frac{\partial g}{\partial a}(A,M) + \bar{m}\frac{\partial g}{\partial m}(A,M), \end{aligned}$$

where $\lambda$ is an eigenvalue and $\bar{a}$, $\bar{m}$ are eigenfunction components; these are all complex-valued. The notation $\frac{\partial f}{\partial a}(A,M)$ (for example) is the derivative of *f* with respect to *a*, evaluated at the travelling wave solution $A(\xi$), $M(\xi )$. Whilst the travelling wave solution of (7) must be periodic by definition, the eigenfunction need not be. That being said, although we pose the problem on $0<\xi <L$, our results must hold on an infinite domain. One can derive appropriate boundary conditions for $\bar{a}$, $\bar{m}$ using Floquet theory (Deconinck and Kutz 2006; Rademacher et al. 2007; Fiedler 2002)—for some real valued constant $\gamma$,

10 $$\begin{aligned} \bar{a}(L)=\bar{a}(0){\text {e}}^{i \gamma },\quad \bar{m}(L)=\bar{m}(0){\text {e}}^{i \gamma }. \end{aligned}$$

This ensures that the complex amplitude of the eigenfunction is the same at both ends of the domain, preventing unbounded growth when applied to the infinite domain case. A phase shift is, however, permissible via the imaginary exponent—because of the coupling in (9) the phase shift must be identical across all real and imaginary parts of the eigenfunction components. This means that for a given travelling wave solution defined on $0<\xi <L$, a perturbation can be characterised by $\gamma$, and we can assess whether it grows or decays by studying the associated $\lambda$. It now becomes clear that numerical continuation is a powerful way of testing stability since one can track the values of $\lambda$ by using $\gamma$ as a continuation variable.

**Fig. 3:**
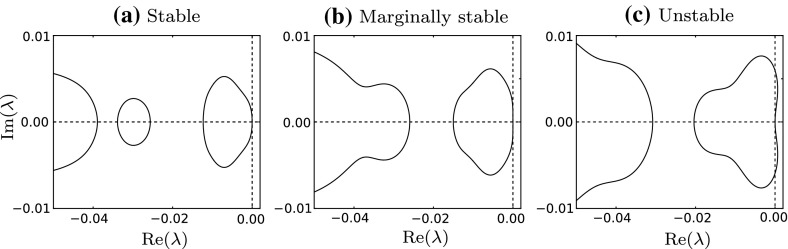
Spectra of the travelling wave solutions of (6) with $\nu =300$. In **(a)**, $\beta =10$ and the spectrum is contained in the left of the complex $\lambda$ plane indicating that the solution is stable. In **(c)**, $\beta =50$ which corresponds to an unstable solution since there exist perturbations with positive growth rate. In **(b)**, $\beta = 16.22$ and the solution is marginally stable. The transition **(a)**–**(c)** is an illustration of an Eckhaus instability, where destabilisation occurs via a change of curvature at the origin. One can trace out contours of zero curvature in parameter space as in Fig. 5 to mark the boundary between 1D stable and unstable solutions

Stability can be determined by calculating the spectrum; for general spatio-temporal solutions, this will contain both the discrete “point spectrum” and the continuous “essential spectrum”, but in the specific case of travelling wave solutions, the point spectrum is empty (see Chapter 3.4.2 in Fiedler 2002). Therefore, the spectrum is just the essential spectrum given by the set of $\lambda$ values such that (9) with (10) has a non-trivial solution. We plot some spectra of travelling wave solutions in Fig. 3. For all, we observe that the spectrum passes through the origin, which is the case for all travelling wave solutions and reflects the neutral stability of waves to translation. Therefore, we assert that a solution is (spectrally) stable if ${\text {Re}}(\lambda )<0$ for all $\lambda$ except $\lambda =0$ (all perturbations will decay over time), and unstable if $\lambda$ values exist with ${\text {Re}}(\lambda )>0$ (a range of perturbations will grow over time). Points in the complex $\lambda$ plane that satisfy $\max ({\text {Re}}(\lambda ))=0$ (excluding the origin) indicate a marginally stable solution. In Fig. 3a, we have a stable solution, meaning that the corresponding solution of the original PDE model will persist. In many cases, destabilisation is a result of a change of curvature at the origin, and so it is sufficient to examine the spectrum close to the origin. This type of destabilisation mechanism is known as an “Eckhaus” or “sideband” instability and is illustrated in Fig. 3. We mention that in general instability can also be of “Hopf” type, meaning that destabilisation occurs away from the origin; however, numerical work suggests that for (1) we need only consider the Eckhaus case.

### 2D stability: the envelope of the spectrum

To motivate this section, we give a brief analogy. Consider learning to ride a bicycle, in particular, the stability of the cyclist. One might start by using stabilisers—this is now a 1D problem and all the cyclist has to worry about is falling forwards or backwards. Hopefully, the cyclist is 1D stable, and can eventually remove their stabilisers, opening them up to a whole new set of perturbations acting in a perpendicular direction to the motion of the bicycle. This is the full 2D problem and though the cyclist may be stable to 1D perturbations, they could be unstable to 2D perturbations, causing them to fall sideways. In this section, we describe the basic framework necessary for 2D stability by using a simple 2D analogue of (9). We then describe how this new equation can be analysed numerically, using (9) as a starting point.

We begin by taking $a=a(x,y,t)$, $m=m(x,y,t)$, and $\varDelta = \partial ^2/\partial x^2 + \partial ^2/\partial y^2$ in (1). Since striped patterns are constant in the *y*-direction, they are solutions of (6) with a trivial redefinition $A(\xi , y)$, $M(\xi ,y)$. In contrast, to determine stability we must assume the solution is non-constant in the *y*-direction due to the addition of small perturbations. Therefore, in the same vein as Sect. 3.1, striped pattern solutions are *t*-independent, *y*-independent solutions of

11a $$\begin{aligned} \frac{\partial \hat{a}}{\partial t}&= \nu \left( \frac{\partial ^2 \hat{a}}{\partial \xi ^2} + \frac{\partial ^2 \hat{a}}{\partial y^2} \right) + (c+\beta )\frac{\partial \hat{a}}{\partial \xi } + f(\hat{a},\hat{m}), \end{aligned}$$

11b $$\begin{aligned} \frac{\partial \hat{m}}{\partial t}&= \frac{\partial ^2 \hat{m}}{\partial \xi ^2} + \frac{\partial ^2 \hat{m}}{\partial y^2} + c\frac{\partial \hat{m}}{\partial \xi } + g(\hat{a},\hat{m}), \end{aligned}$$

where $\hat{a}=\hat{a}(\xi ,y,t)$, $\hat{m}=\hat{m}(\xi ,y,t)$. Like the periodic travelling waves considered in Sect. 3.1, striped patterns are periodic in the *x*-direction and perturbations must be represented by a general eigenfunction equation. However, because of the homogeneity in the *y*-direction, one can decompose the eigenvector (see Sect. 2), and represent corresponding perturbations using the wavenumber, $\ell$. Thus we perturb the striped pattern solution as

12 $$\begin{aligned} \hat{a}(\xi ,y,t)=A(\xi ,y) + \bar{a}(\xi ){\text {e}}^{i\ell y + \lambda t},\quad \hat{m}(\xi ,y,t)=M(\xi ,y) + \bar{m}(\xi ){\text {e}}^{i\ell y + \lambda t}, \end{aligned}$$

for small $\bar{a}(\xi )$, $\bar{m}(\xi )$. The linear eigenvalue problem is obtained by substituting (12) into (11) and linearising about the striped pattern solution, giving,

13a $$\begin{aligned} \lambda \bar{a}&= \nu \left( \frac{\partial ^2 \bar{a}}{\partial \xi ^2} - \ell ^2 \bar{a} \right) + (c+\beta )\frac{\partial \bar{a}}{\partial \xi } + \bar{a}\frac{\partial f}{\partial a}(A,M) + \bar{m}\frac{\partial f}{\partial m}(A,M), \end{aligned}$$

13b $$\begin{aligned} \lambda \bar{m}&= \frac{\partial ^2 \bar{m}}{\partial \xi ^2} - \ell ^2 \bar{m} + c\frac{\partial \bar{m}}{\partial \xi } + \bar{a}\frac{\partial g}{\partial a}(A,M) + \bar{m}\frac{\partial g}{\partial m}(A,M). \end{aligned}$$

This is simply a generalisation of (9): when $\ell =0$, perturbations are constant in the *y*-direction, and the problem is equivalent to the 1D case already considered in (9). These inherently 1D perturbations now work in tandem with a heterogeneity in the transverse direction when $\ell \ne 0$, with certain pairings having a possible positive growth rate, leading to destabilisation of the solution. The aim now is to determine which combination of 1D and 2D perturbations has the maximum ${\text {Re}}(\lambda )$—if this maximum is negative, we can conclude that the solution is stable in both 1D and 2D, otherwise the solution is either 1D stable and 2D unstable, or, unstable in both 1D and 2D. We now outline a numerical algorithm that can be used to test stability.

#### Outline of numerical computation

To begin with, we rewrite the equations described in previous sections for numerical continuation—all our continuations are implemented using AUTO 07p (Doedel et al. 2007), for which equations must be in the form $u'=H(u)$. We are not aware of any publications detailing this calculation, though related work is described by Siero et al. (2015). We can easily write (6) and (13) as first order systems, respectively:

14 $$\begin{aligned}&\left. \begin{aligned} \frac{\text {d} A}{\text {d} \xi }&= B,\quad \frac{\text {d} M}{\text {d} \xi } = N \\ \frac{\text {d} B}{\text {d} \xi }&= -\frac{1}{\nu }\left( (c+\beta )B + f(A,M) \right) , \\ \frac{\text {d} N}{\text {d} \xi }&= -\left( cN + g(A,M) \right) , \end{aligned} \right\} \end{aligned}$$

15 $$\begin{aligned}&\left. \begin{aligned} \frac{\text {d} \bar{a}}{\text {d} \xi }&= \bar{b},\quad \frac{\text {d} \bar{m}}{\text {d} \xi } = \bar{n}, \\ \frac{\text {d} \bar{b}}{\text {d} \xi }&= \frac{1}{\nu }\left( \lambda \bar{a} + \ell ^2 \bar{a} - (c+\beta )\bar{b} - \bar{a}\frac{\partial f}{\partial a}(A,M) - \bar{m}\frac{\partial f}{\partial m}(A,M) \right) , \\ \frac{\text {d} \bar{n}}{\text {d} \xi }&= \lambda \bar{m} + \ell ^2 \bar{m} - c\bar{n} - \bar{a}\frac{\partial g}{\partial a}(A,M) - \bar{m}\frac{\partial g}{\partial m}(A,M), \end{aligned} \right\} \end{aligned}$$

with boundary conditions calculated from (10). Note that AUTO 07p does not allow for the continuation of complex variables, meaning that (15) corresponds to eight equations when one takes the constituent real and imaginary parts.

**Fig. 4:**
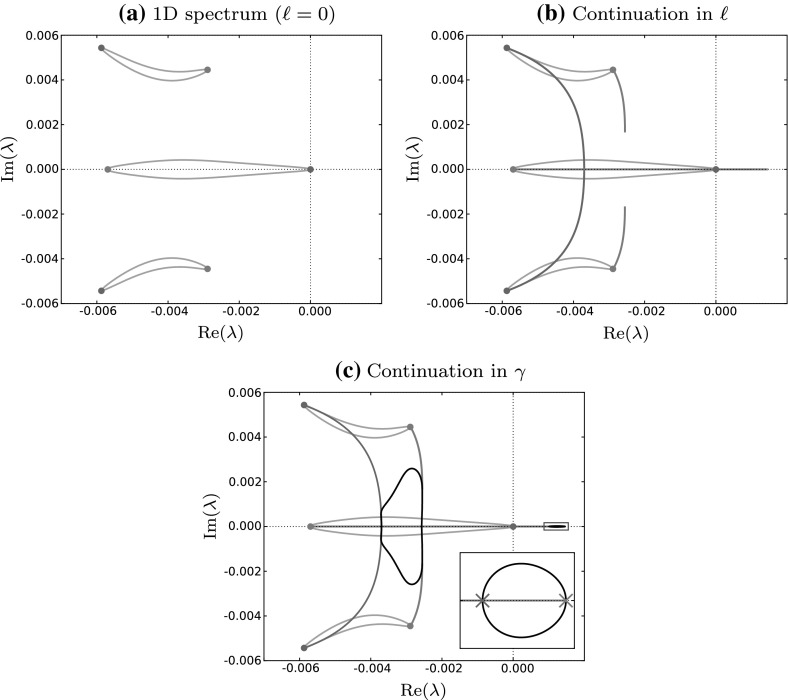
Panels show the numerical continuation methodology for testing stability in two space dimensions using (14)–(16). In **(a)**, we plot the spectrum of a 1D stable solution ($\ell =0$) in grey, and mark the points where $\gamma =0$ (blue circles) and $\gamma =\pi$ (red circles). In **(b)**, we increase the $\ell$ parameter (coloured curves) from zero to include transverse 2D perturbations, and search for turning points with ${\text {Re}}(\lambda _l)=0$—saving the solution with $\max {\text {Re}}(\lambda )$. In **(c)**, we trace out contours of maxima by imposing the condition, ${\text {Re}}(\lambda _l)=0$, and continuing in $\gamma$. This contour is the envelope (black curve) of the 1D spectrum and represents the most unstable combinations of 1D and 2D perturbations. Because the original 1D spectrum in **(a)** is contained in the left half of the complex plane, but a section of the envelope overlaps into the right, we conclude that the solution is 1D stable, 2D unstable. The inset in **(c)** is a blow up of the region ${\text {Re}}(\lambda ) \in [0.0009, 0.0015]$, ${\text {Im}}(\lambda )\in [-\,0.00003,0.00003]$. The red and blue crosses represent the two main destabilisation mechanisms and correspond to the marginal stability curves of the same colours in Fig. 5 (colour figure online)

Parameters can be numerically continued, but the method first requires an initial solution from which to start the computation. To find one, we note that for $\gamma =0$ (9) can be discretised in $\xi$ and written as a matrix eigenvalue problem. Standard numerical techniques can then be implemented to obtain a discrete set of approximated eigenvalues. For stability, we are only interested in the eigenvalues that have the largest ${\text {Re}}(\lambda )$. Sorting the numerically computed eigenvalues with respect to ${\text {Re}}(\lambda )$ and choosing the largest 10 (say), together with their corresponding eigenvectors, gives us a set of starting points for continuation. The blue points in Fig. 4a are our initial eigenvalues with $\gamma =0$, and using the method of Rademacher et al. (2007), continuation of each of these points in $0< \gamma < 2\pi$ allows us to “fill in the gaps”, and trace out the full spectrum.

The reason one must perform a continuation from each initial $\lambda$ is that the spectrum is often not made up of one continuous curve; instead, it consists of several branches. In fact, isolated islands of spectrum are common (see Fig. 4a), especially near the critical region for the determination of stability. Consequently, we are assuming the following: for every disconnected subset of spectrum, there exists at least one eigenvalue with $\gamma =0$ contained within it. In principle, this assumption might not hold, however, we are not aware of any examples where this is not the case and in simpler systems the existence of such islands has been disproved (Rademacher et al. 2007).

We can now calculate the spectrum for any fixed $\ell$, and the starting point for 2D stability is the spectrum that determines 1D stability, for which $\ell = 0$. Suppose we have a 1D stable solution; one could generate spectra for different fixed values of $\ell$ until one finds a perturbation that destabilises the solution. It can then be concluded that this specific pattern is 1D stable, 2D unstable. Aside from this being a long and tedious process, a conclusion cannot be drawn about 2D stability unless one can determine such a value of $\ell$. Instead, we must be able to calculate the most unstable point of the spectrum, over all values of $\ell$. We implement this idea by letting $\bar{a}$, $\bar{m}$, $\bar{b}$, $\bar{n}$ and $\lambda$ be dependent on both $\gamma$ and $\ell$. Then we can consider the quantity $\lambda _l := \partial \lambda / \partial \ell$ through a third set of equations:

16 $$\begin{aligned} \left. \begin{aligned} \frac{\text {d} \bar{a}_l}{\text {d} \xi }&= \bar{b}_l,\quad \frac{\text {d} \bar{m}_l}{\text {d} \xi } = \bar{n}_l,\\ \frac{\text {d} \bar{b}_l}{\text {d} \xi }&= \frac{1}{\nu } \bigg ( \lambda _l \bar{a} + \lambda \bar{a}_l + 2 \ell \bar{a} + \ell ^2 \bar{a}_l - (c+\beta )\bar{b}_l \\&\quad - \bar{a}_l\frac{\partial f}{\partial a}(A,M) - \bar{m}_l\frac{\partial f}{\partial m}(A,M) \bigg ), \\ \frac{\text {d} \bar{n}_l}{\text {d} \xi }&= \lambda _l \bar{m} + \lambda \bar{m}_l + 2 \ell \bar{m} + \ell ^2 \bar{m}_l - c\bar{n}_l\\&\quad - \bar{a}_l\frac{\partial g}{\partial a}(A,M) - \bar{m}_l\frac{\partial g}{\partial m}(A,M), \end{aligned} \right\} \end{aligned}$$

that are obtained by differentiating (15) with respect to $\ell$—subscripts denote partial differentiation. Together with (14) and (15), and noting again that (16) contains complex quantities, this gives us a set of twenty real equations in total to be used in the continuation. The required boundary conditions for (16) can be obtained by differentiating (10) with respect to $\ell$.

We begin by picking a starting point on the 1D spectrum corresponding to a specific 1D perturbation. These points are represented in Fig. 4a as coloured circles. From a practical point of view, a fundamental function of AUTO 07p is the ability to detect and save user defined restart information from which a subsequent continuation can be done. We perform continuations in the $\ell$ parameter for fixed $\gamma$, and look for turning points by detecting solutions with ${\text {Re}}(\lambda _\ell ) = 0$. Of the turning points, the value of $\lambda$ with the largest real part must be saved as a new starting point for the next stage in the algorithm. Of course, for cases where there is more than one maximum, care must be taken to select the largest. This is a particular issue in the neighbourhood of the origin.

A visual representation of these $\ell$ continuations can be seen in Fig. 4b as coloured curves, the end points of which represent the most unstable point for each fixed $\gamma$. If no maximum is detected, one must assume the maximum occurs at $\ell =0$. Once a maximum is determined, we can fix ${\text {Re}}(\lambda _\ell ) = 0$, and trace out the envelope of the spectrum with a continuation in $\gamma$, which is illustrated in Fig. 4c. If the envelope overlaps into the right hand half of the $\lambda$ complex plane, this implies that a range of perturbations with a transverse heterogeneity are the source of instability. This is the case in Fig. 4c and we can conclude that the solution is 1D stable and 2D unstable. If the envelope is contained within the left half of the $\lambda$ complex plane, we have stability against both 1D and 2D perturbations, meaning the striped pattern will persist.

**Fig. 5:**
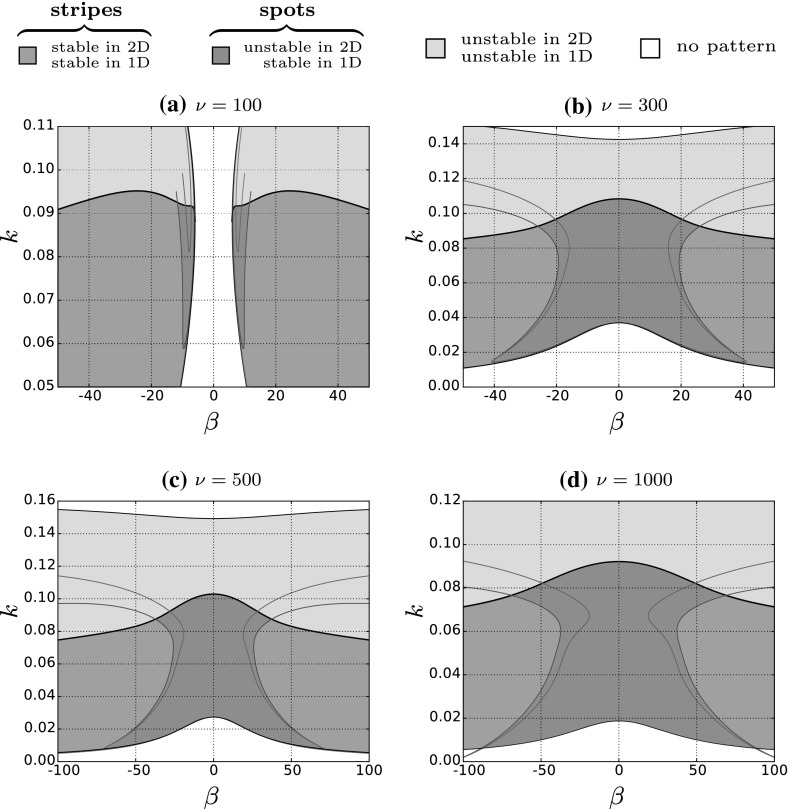
Stability of existing striped patterns in young mussel beds. Striped pattern solutions of (1) are represented in terms of their tidal flow rate, $\beta$, and their wavenumber, *k*, as a pair in $(\beta ,k)$ parameter space for different algal dispersal rates, $\nu$. The thin black curves are Turing–Hopf bifurcation loci that bound the pattern forming region (union of all coloured sub-regions). The thick black line is the Eckhaus curve that separates 1D stable patterns (union of green and dark grey sub-regions) from 1D unstable patterns (light grey sub-region). Patterns unstable in 1D are also unstable in 2D. The red/blue curves form the basis of this paper, and partition the 1D stable region into 2D stable (green) and 2D unstable (dark grey) sub-regions. Striped patterns in the dark grey region destabilise and form spotted patterns (colour figure online)

## Results and simulation

The envelope of the spectrum (see Sect. 3) allows one to calculate the most unstable 2D perturbation for any given striped pattern, from which one can infer stability. For each fixed $\beta$ a range of stable solutions may exist that can be characterised by their wavenumber. Previous work (Rademacher et al. 2007; Sherratt 2012, 2013b) has detailed how one can map out marginal stability boundaries associated with 1D solutions in parameter space. In this section we explore how the flow rate affects the 2D stability of striped patterns by mapping stable solutions in the $(\beta ,k)$ parameter plane, where *k* is the wavenumber of the initial striped pattern. To determine the 2D stable patterns we first must consider the set of 1D stable patterns, and so in Fig. 5 we trace out the Eckhaus marginal stability curve that separates 1D stable and unstable patterns. We now must partition the 1D stable region into 2D stable and unstable regions.

The calculation of the 1D (Eckhaus) boundary is dependent on the fact that all spectra of travelling waves pass through the origin of the complex $\lambda$ plane. In contrast, for 2D stability we consider the envelope where this is not necessarily the case (see Fig. 4c). Suppose we calculate the envelope for a particular stripe pattern solution—this will tell us the stability of that solution for a particular fixed $\beta$. Iterating this process with a gradual change in $\beta$ will slowly alter the shape and position of the envelope until we obtain a solution that is marginally stable to 2D perturbations, i.e. $\max ({\text {Re}}(\lambda ))=0$. This solution marks the boundary between stable and unstable striped patterns and we observe that it occurs at $\lambda =0$, exactly. To be clear, unlike 1D Eckhaus stability where one observes a change in curvature at the origin of the spectrum (see Fig. 3), we find that 2D instability occurs via a translation of the envelope through the origin. More concretely, we find that marginal stability always seems to occur for points on the envelope where either $\gamma = 0$ or $\gamma = \pi$, which has previously been reported (for a different model) by Siero et al. (2015), and in particular the points on the 1D spectrum from which they originate are: $\lambda =0$ ($\gamma =0$), or the value of $\lambda \ne 0$ obtained after one continuation of $\gamma \in [0,\pi ]$ from $\lambda =0$. The consideration of the corresponding points on the envelope alone, which we illustrate in Fig. 4c with coloured crosses, allows us to simplify our calculation considerably. Computationally, we deal with these two points separately. Once we have found the most unstable 2D perturbation for our chosen fixed $\beta$, we impose the condition ${\text {Re}}(\lambda _\ell )=0$ and vary $\beta$ (allowing $\ell$ to vary) until we find a critical value where $\lambda =0$. Finally, continuation in both $\beta$ and *k* with the additional constraints that $\lambda =0$ and $\lambda _\ell =0$ traces out marginal stability boundaries as seen in Fig. 5. We trace out the boundaries for both the $\gamma =0$ and $\gamma = \pi$ cases which separates the 1D stable region into 2D stable and 2D unstable sub-regions.

**Fig. 6:**
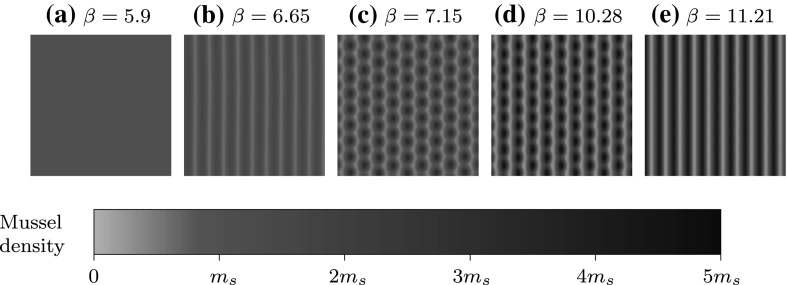
Onset of pattern formation in 2D for $\nu =100$ corresponding to Fig. 5a. We solved (1) on the unit square with periodic boundary conditions. Initially we have the stable homogeneous steady state given by (2) which is perturbed with small amplitude random noise **(a)** that we subject to increasing flow rates by varying $\beta$ at a constant rate of $10^{-5}$. A Turing–Hopf bifurcation exists at $\beta \approx 5.93$, beyond which a striped pattern is generated perpendicular to the direction of advection **(b)** with $k=0.0877$. Note that immediately after onset the pattern is 2D stable but for an insignificantly small range of small amplitude patterns—approximately $\beta \in (5.93,6.18)$. As $\beta$ increases the solution becomes unstable to transverse 2D perturbations and a rhombic spotted pattern is generated (**c**, **d**), though full break up does not occur. A striped pattern of the same wavenumber reforms with larger amplitude (**e**). Note that $m_s$ is the homogeneous steady state defined in (2) which is unchanged as $\beta$ is varied

**Fig. 7:**
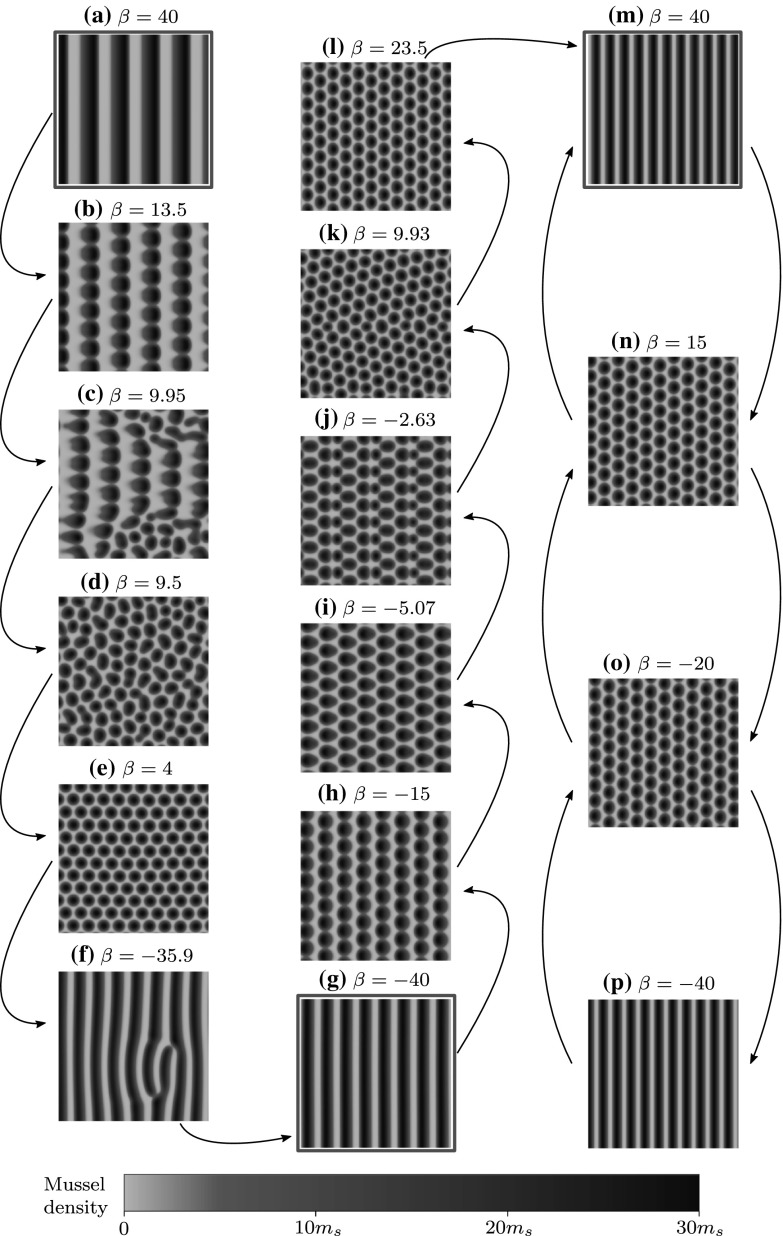
2D hysteresis effects in mussel beds caused by changing tidal currents for $\nu =300$. We solved (1) on the unit square with periodic boundary conditions. Panels are snapshots of a single numerical simulation of (1), where $\beta$ varies at a constant rate of $10^{-4}$ back and forth between $\beta =\pm \,40$. The relevant stability diagram can be seen in Fig. 5b. Initially, we have a pattern with wavenumber $k=0.04$ repeated fivefold (**a**). As the flow weakens, a spotted pattern emerges (**b**), breaks up (**c**), (**d**) and forms a new regular spotted pattern (**e**). As the direction of flow changes and strengthens, a striped pattern begins to reform with defects (**f**) that subsequently disappear leaving a pattern with $k=0.064$ (**g**). As $\beta$ is varied from − 40 to 40 we see spots (**h**) that become distorted into droplet shapes (**i**) and destabilise (**j**), (**k**) into a different spotted pattern (**l**). This pattern reforms into a striped pattern with $k=0.088$ (**m**). Repeating the process using the solution in **(m)** as a starting point generates spotted patterns like those in **(n)**, **(o)**, but the reformed striped pattern (**p**) is identical to that in **(m)**. Note that in the absence of history dependence one would expect the same patterns for $\pm \,\beta$ due to symmetry in (1). Therefore, the red boxed panels demonstrate three distinct striped patterns for, essentially, the same flow rate

Through numerical simulation of (1) we find that perturbed 1D stable, 2D unstable stripe solutions breakup to form regular spotted patterns. The striped solution is periodic and stable in the *x* direction, and homogeneous but unstable in the *y* direction, with the instability (similar to onset discussed in Sect. 2) inducing an additional periodicity in the *y* direction. The two $\gamma$–destabilisation mechanisms initiate two distinct types of break up of stripes. For $\gamma = 0$ ‘square’ break-up occurs meaning that spots align in both the *x* and *y* directions. For $\gamma = \pi$ ‘rhombic’ break-up occurs which generates a spotted pattern where columns of spots are out of phase in the *x* direction; this is visible in Figs. 6c and 7b, h and n, for example. Tracing out the $\gamma$-curves reveals that for our chosen parameter set the primary break up mechanism is almost always the $\gamma = \pi$ curve (giving rhombic patterns); this is the curve that (almost always) bounds the 2D unstable region in Fig. 5. This is confirmed in numerical simulations of (1), which also reveals that rhombic break up is the dominant mechanism if both destabilisation criteria are met. For very large wavelength stripes the curves briefly intersect in Fig. 5b and c, and the $\gamma =0$ destabilisation mechanism becomes relevant, presenting the opportunity for square break up; however the relevant region for spotted patterns is insignificantly small and corresponds to very weakly unstable solutions. Numerical simulations in this region do generate faint square 2D patterns, but full break up never occurs so that spots are not seen in practice.

In Fig. 5 we find that the 2D unstable region is always present at comparatively low flow rates, implying that stripe break up into spots may be a significant process in mussel beds when considering the oscillatory nature of the tide. A realistic rate of algal dispersal is difficult to determine, in part due to its obvious simplification of algal movement. Cangelosi et al. (2015) argue the rough estimate $\nu = 300$, though there is no concrete evidence to support this. In this regard, we assess the effect that algal dispersal has on stability by considering a few different values of $\nu$. When algal dispersal is low and a critical flow rate for pattern formation must be achieved (see Fig. 5a), patterns are 2D stable immediately after onset, but this region is too small to be relevant in real mussel beds. When the algal dispersal rate is increased patterns are generated for all flow rates, however the 2D unstable region becomes larger. Increasing $\nu$ makes striped pattern formation more likely, but also increases the critical tidal flow rate for which the patterns will be resilient to transverse disturbances, making spotted patterns relevant for a wider range of flow rates. We conclude from Fig. 5 that spotted patterns are a consequence of low tidal flow rates, and the persistence of striped patterns requires higher rates of flow than previously expected. We highlight a key point: although the 2D stability boundaries in Fig. 5 can be used to determine when stripes become spots, they do not apply to the converse situation—i.e. a spotted pattern that is subject to an increased rate of flow may persist into the 2D stable stripe region.

Figure 6 shows a numerical simulation for a relatively low rate of algal dispersal, and pattern formation does not occur until a critical flow rate is reached. Suppose the mussel bed is initially at its homogeneous steady state given by (2), and the flow rate begins to increase from $\beta =0$ as in Fig. 6. For flow rates below a critical value $\beta =\beta _0$ the steady state is stable, but for $\beta >\beta _0$ stripes begin to form. Immediately after onset we find that striped patterns are 2D stable, though as mentioned for a very limited range of $\beta$ values. Increasing $\beta$ further still, stripes become unstable to transverse 2D perturbations giving rise to spotted patterns, before the flow becomes strong enough for spots to reform into stripes.

We investigated history dependence in mussel beds by using Fig. 5b to inform our numerical simulations. Figure 7 shows the results of a simulation in which we slowly oscillated $\beta$ between maximum and minimum flow rates at a constant rate, which reveals that a number of distinct striped patterns can exist for the same $\beta$. This hysteresis effect is novel due to the fact that transitions are purely a result of transverse instabilities and are consequently unreported in the literature. If one considered a 1D treatment of the problem resulting in the Eckhaus curve alone, one would conclude that the transformation from one striped pattern to another would be a consequence of high flow rates. In contrast, our results provide a more relevant destabilisation mechanism when considering the transition between flood and ebb currents during a period of tidal oscillation. Figure 7 also demonstrates that spotted patterns themselves are not necessarily stable; the spotted pattern in Fig. 7b breaks up, and a new spotted pattern emerges in Fig. 7e.

## Ecological implications and discussion

We have analysed an extended reduced losses model (1) for striped mussel beds that was originally posited in two space dimensions (van de Koppel et al. 2005). Nonetheless, much of the mathematical analysis has focused on the one dimensional case; assuming results can be applied trivially in 2D. The one dimensional solutions are periodic travelling waves which we extend in two space dimensions as stripe patterns, and analyse using numerical continuation techniques and simulation. Specifically we have examined how the tidal flow rate affects the resilience of stripes, and we summarise the ecological implications as follows.

(i) Our main result is that large scale spotted patterns in mussel beds are a consequence of low tidal flow rates. Once a striped pattern has formed, a critical minimum flow rate must be attained for ecological resilience, otherwise, the striped pattern is an effective transitional phase in the formation of spotted patterns. An ecologist interested in determining resilient striped patterns should note that $\beta$ must be stronger than previously thought in this regard. If not, stripes will break up and a patchy appearance of the mussel bed may be observed in practice, as seen in Fig. 1. The authors in Siero et al. (2015) determined that striped vegetation patterns in semi-deserts were more resilient on steeper slopes (an equivalent advection coefficient to $\beta$ is used to increase the flow rate of water down the slope); in this regard our results are in correspondence.
(ii) A higher rate of algal dispersal in the lower water layer permits the generation of periodic stripe patterns at lower flow rates, though additionally it encourages their break up. Although the model we consider incorporates a simple approximation of true algal movement, we can still hypothesise what physical attributes of the system might influence $\nu$. The random movement of algae is determined by complex mixing processes in the ocean caused by turbulence—primarily on the millimetre scale. Physical properties of the ecosystem that might influence these processes in intertidal regions include: temperature, roughness of the seabed and wave action (Dower et al. 1997).
(iii) We have identified a new type of hysteresis affect in mussel beds that is a result of small disturbances perpendicular to the direction of tidal flow. Building on previous work (Sherratt 2013b), our consideration of transverse 2D perturbations of stripes has revealed new destabilisation mechanisms which cause their break up. With guidance from the stability map in Fig. 5 we simulated (1) numerically for slowly varying $\beta$ in two space dimensions and find that transitions between distinct striped patterns occur as a consequence of the 2D instability. Each striped pattern is dependent upon the previous state of the system.

The factors mentioned in (ii) that influence the dispersal of algae occur concurrently to generate eddies that affect the mixing of algae in a complicated way, the variation of which is crudely reflected in (1) with a change in algal dispersal rate. Due to the obvious model simplification $\nu$ is very difficult to estimate, therefore we performed our calculations for a range of values. An interesting direction for further work in this regard would be to incorporate a more realistic model for the random movement of algae, not only in the *x* and *y* directions, but also between water layers. Experimental work could also aid in the determination of a more informed choice of $\nu$ that could be used in our calculations. Nevertheless, an extension of the original reduced losses model to include a simplistic random movement term for algae is a more accurate representation of the real world problem, and the consideration of a range of dispersal rates leads us to the conclusion set out in (ii).

**Fig. 8:**
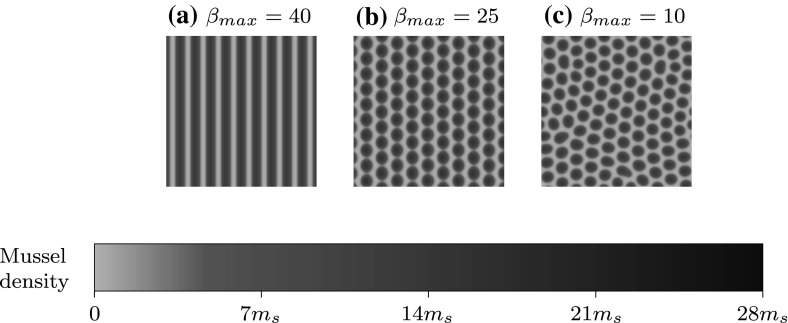
The effects of oscillating tidal flow are shown through simulation of (1) with $\beta =\beta _{max}\cos (2\pi t/T)$ for three different values of $\beta _{max}$. Parameters and domain size are identical to that in Fig. 8 with the exception of $\beta$, and we use Fig. 5b to inform our choices of $\beta _{max}$. We assume a semi-diurnal tide where two tidal oscillations occur per day, which corresponds to the nondimensionalised time period: $T=2000$. Panels are the solutions after 100 tide oscillations (50 days) with the addition of random noise every few time steps. In all cases a pattern with wavenumber $k=0.088$ (identical to that observed in Fig. 7m) emerges quickly. After this: **(a)** stripes persist despite short intervals of low flow rate, **(b)** stripes break up to form a spotted pattern which maintains its structure during subsequent oscillations, **(c)** stripes break up to form a spotted pattern with defects that persist. We solved (1) on the unit square with periodic boundary conditions

The most changeable parameter in (1) is the tidal flow rate, though our analysis has focused on the case where $\beta$ is constant; making our results most relevant when $\beta$ varies slowly. In reality, the flow rate in intertidal regions oscillates much faster and in a more sinusoidal fashion. Furthermore, a unidirectional flow causes a constant collective pattern migration in the opposite direction, though there is no evidence to support this. An oscillatory, bidirectional flow ensures that no net migration occurs (Sherratt 2016). Simulations of (1) for a sinusoidal flow rate with maximum amplitude $\beta _{max}$ reveals that stripes may withstand brief intervals of low flow rate. This is dependent on $\beta _{max}$ which, assuming a constant period of oscillation, affects the rate of change of $\beta$ and the duration that stripes are subject to the low, destabilising flow rates. Figure 8 shows how $\beta _{max}$ affects the long term evolution of stripes when subject to tidal oscillation, and demonstrates how Fig. 5 can be used to roughly gauge the outcome. Note that apart from $\beta$ this simulation is identical to that in Fig. 7 and we find that the same wavelength pattern is generated in Fig. 8a as seen in Fig. 7l. For more rigorous results, further work could focus on performing our analysis on (1) with $\beta =\beta (t)$. Despite this shortcoming in our analysis, we believe that (iii) will still be significant in real mussel beds because of slower tidal variations throughout the year. Of course, there is a regular oscillation of the tidal flow rate during a day, but there are also biweekly spring and neap tides known for their more extreme tide highs and lows that result in larger and smaller $\beta _{max}$ respectively Lalander et al. (2013); to an extent that depends on the geographical context, for instance basin geometry. Additionally, the relative position of the Earth and Moon in their collective elliptic orbit of the Sun gives rise to both abnormally strong perigean and weak apogean currents that occur three or four times annually (Cartwright 1999). This means that a striped pattern that is resilient to an oscillatory flow with a particular $\beta _{max}$ may be susceptible to break up later on in the year because of a change in $\beta _{max}$. One might therefore expect to see a larger proportion of striped mussel beds around the time of a perigean spring tide, and spotted/patchy mussel beds around the time of an apogean neap tide. This slow variation in $\beta _{max}$ presents the opportunity for break up and reformation of stripes and the possibility of observing the history dependence that we have reported.

In general, testing theoretical predictions about mussel beds is certainly more plausible than for many other ecological systems, e.g. spotted patterns in coral reefs (de Paoli et al. 2017), rows of trees in the ribbon forest (Bekker et al. 2009), banded vegetation in semi-arid desert regions (Klausmeier 1999). This is because pattern generation in young mussel beds is relatively fast and small-scale in comparison with the previous examples. Mussel patterns actually occur on multiple spatial scales (Liu et al. 2014) and previous experiments on small-scale mussel patterns have been possible under lab conditions (Van de Koppel et al. 2008). Though harder to implement at the ecosystem scale, recent work (de Paoli et al. 2017) has included the seeding of mussel beds into various initial formations of large and small scale patterns in order to observe how mussel numbers are affected over time—this enabled the authors to validate the theoretical prediction that self–organisation increases the resistance of mussel beds to disturbances. We believe a similar field experiment could be implemented to test (i)—the key feature of this would be to control and measure maximum flow rate. We point out that spotted patterns will be unlikely to form at very low flow rates since the replenishment of algae would be minimal in reality, leading to the breakdown of the model and, hence, of our predictions.

## References

[CR1] Bekker MF, Clark JT, Jackson M (2009). Landscape metrics indicate differences in patterns and dominant controls of ribbon forests in the Rocky Mountains, USA. Appl Veg Sci.

[CR2] Cangelosi RA, Wollkind DJ, Kealy-Dichone BJ, Chaiya I (2015). Nonlinear stability analyses of turing patterns for a mussel–algae model. J Math Biol.

[CR3] Capelle JJ, Wijsman JW, Schellekens T, van Stralen MR, Herman PM, Smaal AC (2014). Spatial organisation and biomass development after relaying of mussel seed. J Sea Res.

[CR4] Cartwright DE (1999). Tides: a scientific history.

[CR5] Christensen HT, Dolmer P, Hansen BW, Holmer M, Kristensen LD, Poulsen LK, Stenberg C, Albertsen CM, Støttrup JG (2015). Aggregation and attachment responses of blue mussels, Mytilus edulis—impact of substrate composition, time scale and source of mussel seed. Aquaculture.

[CR6] Cox SM, Matthews PC (2002). Exponential time differencing for stiff systems. J Comput Phys.

[CR7] Dame R, Dankers N, Prins T, Jongsma H, Smaal A (1991). The influence of mussel beds on nutrients in the Western Wadden Sea and Eastern Scheldt estuaries. Estuaries.

[CR8] Deconinck B, Kutz JN (2006). Computing spectra of linear operators using the Floquet–Fourier–Hill method. J Comput Phys.

[CR9] de Paoli H, van der Heide T, van den Berg A, Silliman BR, Herman PM, van de Koppel J (2017) Behavioral self-organization underlies the resilience of a coastal ecosystem. In: Proceedings of the national academy of sciences, p 201619203 10.1073/pnas.1619203114 PMC5544259 28696313

[CR10] Dobretsov SV (1999). Effects of macroalgae and biofilm on settlement of blue mussel (*Mytilus edulis* L.) larvae. Biofouling.

[CR11] Doedel EJ, Fairgrieve TF, Sandstede B, Champneys AR, Kuznetsov YA, Wang X (2007) Auto-07p: continuation and bifurcation software for ordinary differential equations. http://citeseerx.ist.psu.edu/viewdoc/summary?doi=10.1.1.423.2590

[CR12] Dolmer P (2000). Algal concentration profiles above mussel beds. J Sea Res.

[CR13] Dower JF, Miller TJ, Leggett WC (1997). The role of microscale turbulence in the feeding ecology of larval fish. Adv Mar Biol.

[CR14] Fiedler B (2002). Handbook of dynamical systems.

[CR15] Gascoigne JC, Beadman HA, Saurel C, Kaiser MJ (2005). Density dependence, spatial scale and patterning in sessile biota. Oecologia.

[CR16] Ghazaryan A, Manukian V (2015). Coherent structures in a population model for mussel–algae interaction. SIAM J Appl Dyn Syst.

[CR17] Guiñez R, Castilla JC (1999). A tridimensional self-thinning model for multilayered intertidal mussels. Am Nat.

[CR18] Holzer M, Popović N (2017). Wavetrain solutions of a reaction–diffusion–advection model of mussel–algae interaction. SIAM J Appl Dyn Syst.

[CR19] Hughes R, Griffiths C (1988). Self-thinning in barnacles and mussels: the geometry of packing. Am Nat.

[CR20] Klausmeier CA (1999). Regular and irregular patterns in semiarid vegetation. Science.

[CR21] Lalander E, Thomassen P, Leijon M (2013). Evaluation of a model for predicting the tidal velocity in Fjord entrances. Energies.

[CR22] Liu QX, Weerman EJ, Herman PM, Olff H, van de Koppel J (2012) Alternative mechanisms alter the emergent properties of self-organization in mussel beds. In: Proceedings of the royal society, The Royal Society, London, p rspb20120157 10.1098/rspb.2012.0157 PMC3367779 22418256

[CR23] Liu QX, Herman PM, Mooij WM, Huisman J, Scheffer M, Olff H, Van De Koppel J (2014). Pattern formation at multiple spatial scales drives the resilience of mussel bed ecosystems. Nat Commu.

[CR24] Newell RI (1989) Species profiles: life histories and environmental requirements of coastal fishes and invertebrates (north and mid-atlantic): blue mussel. Tech. rep., Army Engineer Waterways Experiment Station, Vicksburg, MS (USA); National Wetlands Research Center, Slidell, LA (USA); Maryland Univ., Cambridge, MD (USA). Horn Point Environmental Labs

[CR25] Øie G, Reitan KI, Vadstein O, Reinertsen H, Vadstein O, Olsen Y (2002). Effect of nutrient supply on growth of blue mussels (*mytilus edulis*) in a landlocked bay. Sustainable increase of marine harvesting: fundamental mechanisms and new concepts.

[CR26] Okamura B (1986). Group living and the effects of spatial position in aggregations of *Mytilus edulis*. Oecologia.

[CR27] Rademacher J, Sandstede B, Scheel A (2007). Computing absolute and essential spectra using continuation. Physica D.

[CR28] Sherratt JA (2012). Numerical continuation methods for studying periodic travelling wave (wavetrain) solutions of partial differential equations. Appl Math Comput.

[CR29] Sherratt JA (2013). Generation of periodic travelling waves in cyclic populations by hostile boundaries. Proc R Soc Lond Ser A.

[CR30] Sherratt JA (2013). History-dependent patterns of whole ecosystems. Ecol Complex.

[CR31] Sherratt JA (2016). Invasion generates periodic traveling waves (wavetrains) in predator-prey models with nonlocal dispersal. SIAM J Appl Math.

[CR32] Sherratt JA, Mackenzie JJ (2016). How does tidal flow affect pattern formation in mussel beds?. J Theor Biol.

[CR33] Siero E, Doelman A, Eppinga MB, Rademacher JDM, Rietkirk M, Siteur K (2015). Striped pattern selection by advective reaction-diffusion systems: resilience of banded vegetation on slopes. Chaos.

[CR34] Tyler-Walters H (2008) Mytilus edulis. Common mussel. Marine life information network: biology and sensitivity key information sub-programme. Plymouth Marine Biological Association of the United Kingdom

[CR35] van de Koppel J, Rietkerk M, Dankers N, Herman PM (2005). Scale-dependent feedback and regular spatial patterns in young mussel beds. Am Nat.

[CR36] Van de Koppel J, Gascoigne JC, Theraulaz G, Rietkerk M, Mooij WM, Herman PM (2008). Experimental evidence for spatial self-organization and its emergent effects in mussel bed ecosystems. Science.

[CR37] Wang RH, Liu QX, Sun GQ, Jin Z, van de Koppel J (2009). Nonlinear dynamic and pattern bifurcations in a model for spatial patterns in young mussel beds. J R Soc Interface.

